# The impact of physical exercise on mental health and the relationship among physical exercise, emotional regulation and suicidal ideation in Chinese medical students

**DOI:** 10.3389/fpsyg.2025.1609415

**Published:** 2025-08-12

**Authors:** Yiqi Zhang, Junlin He, Han Ruan, Pin Yao, Guoliang Ma

**Affiliations:** ^1^First Affiliated Hospital of Jinzhou Medical University, Jinzhou, China; ^2^School of Health Management, China Medical University, Shenyang, China; ^3^School of Medical Humanities, China Medical University, Shenyang, China; ^4^Department of Health Management, Shenyang Women’s and Children’s Hospital, Shenyang, China; ^5^Nanjing Center for Disease Control and Prevention, Nanjing, China

**Keywords:** physical exercise, medical students, mental health, medical education, suicide ideation

## Abstract

**Background:**

This study aims to investigate the mediating role of emotional regulation in the relationship between physical exercise and suicidal ideation among Chinese medical students. The study specifically examined how exercise-induced enhancement of emotional regulatory capacity may mitigate suicide risk through neurobiological and psychological pathways.

**Methods:**

A cross-sectional study assessed 852 medical students using the Physical Activity Rating Scale-3 (PARS-3) to categorize exercise intensity (low: 51.9%; moderate: 25.9%; high: 22.2%). Suicidal ideation was measured with the Positive and Negative Suicide Ideation Scale (PANSI), emotional regulation capacity with the Emotion Regulation Questionnaire (ERQ), and psychological symptoms with the Symptom Checklist-90 (SCL-90). Independent t-tests, ANOVA tests, and mediation modeling were used for analysis.

**Results:**

The prevalence of suicidal ideation was 8.10% (69/852), consistent with national epidemiological data. Males exhibited significantly lower depression (Δ = −1.24, *p* < 0.05) and anxiety (Δ = −0.87, *p* < 0.05) scores than females. Students engaging in high/moderate-intensity exercise demonstrated lower rates of depression and psychosis compared to low-intensity exercisers (*p* < 0.05). Emotional regulation mediated 38.1% of the protective effect of exercise against suicidal ideation (*p* < 0.01), aligning with established neurobiological pathways linking physical activity to mood regulation.

**Conclusion:**

The physical exercise habits of medical students are intricately linked to their mental and emotional well-being. It is recommended that medical institutions intensify efforts to promote physical exercise and encourage greater participation among medical students. This proactive approach can contribute to reducing suicidal ideation among medical students and enhancing their overall mental health.

## Introduction

Medical students globally face a disproportionate mental health burden characterized by elevated rates of depression, burnout, and suicidal ideation, driven by intense academic pressure, clinical responsibilities, and competitive environments (Taylor et al., 2022; Dyrbye et al., 2017; Suraj et al., 2021). In China, the suicide rate among medical college students surpasses that of their peer groups by a staggering 2 to 4 times, and this grim statistic continues to escalate year by year (Seo et al., 2021). Within this high-risk population, Chinese medical students encounter additional challenges, characterized by prolonged and demanding education, often leave them with limited opportunities for physical exercise (Wallin and Runeson, 2003) and including intense societal expectations, prolonged training periods, and cultural stigma surrounding mental health disclosure, which may exacerbate psychological distress and impede help-seeking behaviors (Li et al., 2024). Alarmingly, studies indicate that the prevalence of depressive symptoms and suicidal ideation among Chinese medical students significantly exceeds that of the general population and non-medical peers (Li et al., 2024). This crisis not only compromises individual well-being but also threatens patient care quality and workforce sustainability.

Physical activity (PA) represents a promising, modifiable protective factor against psychological distress. Systematic reviews confirm that regular PA, particularly moderate-to-vigorous physical activity (MVPA), is consistently associated with reduced burnout, lower depression scores, and enhanced quality of life among medical students globally, with evidence suggesting a dose–response relationship (Gross, 2015). Proposed mechanisms include PA’s role in improving self-efficacy, enhancing self-rated health, and modulating physiological stress responses (Malmborg et al., 2017). Furthermore, higher-intensity PA demonstrates significantly stronger associations with reduced stress compared to lower-intensity activities (Lee et al., 2022). However, despite these benefits, PA engagement among medical students remains suboptimal. Research reveals a significant gap between students’ recognition of PA’s benefits and actual participation, with time constraints and academic workload cited as major barriers (Alnawwar et al., 2023). Crucially, Chinese medical students exhibit strikingly low levels of HEPA (Li et al., 2024), potentially missing out on the most potent mental health benefits.

Existing empirical evidence has underscored the close connection between emotional dysregulation and suicidal ideation (Chen et al., 2021; Shen et al., 2017). Individuals facing intense negative emotions and lacking adaptive emotional regulation strategies are more susceptible to suicidal thoughts. However, the precise mechanisms through which physical activity mitigates suicidal ideation remain elusive. An investigation conducted among medical students in the United Kingdom during the COVID-19 pandemic revealed that daily physical exercise was associated with better mental health outcomes compared to those who did not engage in regular physical activity (Coyle et al., 2021). Given the persistent challenges and uncertainties confronting medical students in their education and future careers, the importance of bolstering their mental health education cannot be overstated. Yet, research on enhancing physical exercise and improving mental health education specifically for medical students remains limited.

In this backdrop, our study takes a substantial sample of Chinese medical students as its focal point, aiming to elucidate the current landscape of physical exercise and the prevalence of suicidal ideation among this demographic (Shen et al., 2017). Additionally, we explore the intricate interrelationships between physical exercise, emotional regulation, and suicidal ideation. This research seeks to provide a theoretical foundation for the prevention and intervention of suicidal ideation among medical students.

Our study addresses several key research questions: What is the relationship between physical exercise, suicidal ideation, and emotional regulation among medical students? Does a correlation exist between physical activity and mental health outcomes, especially regarding suicidal ideation? Lastly, does emotional regulation mediate the relationship between physical exercise and suicidal ideation within this population? Building upon prior research, we hypothesize that physical exercise can mitigate suicidal ideation by enhancing individuals’ emotional regulation abilities (Li et al., 2012). The primary objective of this study is to assess the current state of physical exercise and suicidal ideation in Chinese medical students while investigating the intricate connections between physical activity, suicidal ideation, and emotional regulation in medical students.

## Materials and methods

### Study design

This study employed a questionnaire survey method to collect data from 860 medical students enrolled at a university in Liaoning Province, China. The primary objective was to investigate the current status of physical exercise and suicidal ideation among medical students. To achieve this, the following scales were utilized: Symptom Checklist 90 (SCL-90), Physical Activity Registration Scale-3 (PARS-3), Positive and Negative Suicide Ideation (PANSI) scale, and the Emotion Regulation Questionnaire-Short (ERQ-S). The collected data were subsequently subjected to analysis to explore the relationships between emotional regulation and suicidal ideation within the medical student population. This analysis aimed to shed light on the potential influence of emotional regulation on suicidal ideation, serving as a theoretical basis for the development of prevention and intervention strategies tailored to reduce suicidal ideation among medical students, thereby enhancing their mental well-being.

### Scales

#### SCL-90

The mental health status of medical students was assessed using the SCL-90 (Leichsenring et al., 2020). This scale comprises 90 items grouped into nine symptom dimensions (somatization, obsessive-compulsive, interpersonal sensitivity, depression, anxiety, hostility, phobic anxiety, paranoid ideation, and psychoticism). Each item is rated on a 5-point Likert scale (1 = not at all, 5 = extremely), indicating the degree of distress experienced during the preceding week. The Global Severity Index (GSI), calculated as the sum of all 90 item scores divided by 90, serves as a primary indicator of overall psychological distress. According to established Chinese norms (Wang, 1984): A GSI ≤ 1.50 suggests good mental health. A GSI > 1.50 to 2.50 indicates subthreshold mental health. A GSI > 2.50 signifies clinically significant psychological distress.

Additionally, a Total Raw Score (sum of all 90 item scores) ≥ 160 is also commonly used as an indicator raising concerns for potential psychological disorder severity (Wang, 1984).

#### PARS-3

PARS-3, adapted from Stults-Kolehmainen and Sinha (2014) and Zhang et al. (2020), was employed to gauge the physical exercise levels of medical students over a one-month period. Scores were assigned based on intensity, time, and frequency of exercise, with intensity and frequency rated on a scale of 1 to 5 and time on a scale of 0 to 4. Exercise volume was calculated as follows: Exercise Volume = Intensity × Time × Frequency, with scores ranging from 0 to 100, where higher scores indicated greater physical activity. Participants were stratified into three exercise levels: low (≤19 points), moderate (>19 to ≤42 points), and high (>42 points) (Wang and Liu, 2023).

#### PANSI

The PANSI scale (Chen et al., 2021; Chinese version by Osman et al., 1998) evaluates suicide risk through two dimensions: Positive Suicidal Ideation (PSI): 6 items measuring protective factors (problem-solving confidence), with higher raw scores indicating stronger resilience. Negative Suicidal Ideation (NSI): 8 items assessing active suicidal intent (death wishes) (Yang Du et al., 2023). All items use a 5-point scale (1 = Never to 5 = Always). PSI items are reverse-scored, and the total score is derived from NSI raw +PSI reversed, where higher totals (range: 14–70) indicate greater suicide risk.

#### ERQ-S

The ERQ-S, developed by Preece et al. (2023) comprises 6 items rated on a 7-point Likert scale (1 = “strongly disagree” to 7 = “strongly agree”), measuring two core dimensions: cognitive reappraisal and expressive suppression (Preece et al., 2023). Higher scores on this scale indicated a higher level of emotional regulation ability.

### Statistical analyses

All statistical analyses were performed using SPSS 21.0 (IBM Corp., Armonk, NY, USA). Continuous variables were tested for normality via Shapiro–Wilk test. Normally distributed data are expressed as mean ± standard deviation (SD); non-normally distributed data are reported as median (interquartile range, IQR). Statistical differences were analyzed using independent samples t-tests for gender comparisons. One-way ANOVA followed by Scheffe’s *post hoc* tests for comparisons across exercise intensity groups, contingent on verifying homogeneity of variance. Pearson correlation analysis and linear regression were employed to examine relationships between physical exercise and various factors, including mental health, emotional regulation, and suicidal ideation among medical students. A two-way ANOVA was conducted for each SCL-90 dimension to examine main effects of gender and exercise intensity, and their interaction. Tukey HSD post-hoc tests followed significant main effects. The significance level of *p* < 0.05 was considered statistically significant.

### Quality control

The questionnaire was rigorously designed through a comprehensive review of domestic and international literature, ensuring alignment with research objectives and adherence to principles of clarity, logical sequencing, and unambiguous wording. Prior to formal deployment, a pre-investigation involving 50 medical students was conducted to assess feasibility, identify ambiguities, and optimize question structure; this pre-test phase also informed sample size estimation for the main study based on effect size variability observed in pilot data. During the data collation stage, we meticulously reviewed the collected questionnaires. 8 questionnaires exhibiting inconsistencies in responses, a repeated selection of the same option throughout the survey, or patterns of systematically selected options were excluded from the analysis. The questionnaire of SCL-90, PARS-3, PANSI and ERQ-S demonstrated a high level of reliability, with a Cronbach’s *α* coefficient of 0.910, 0.890, 0.895 and 0.880, respectively, underscoring its consistency and dependability. Harman’s one-way test was applied to assess common method bias, revealing that four factors with eigenvalues exceeding 1 were identified. The cumulative variance explained by the first factor was 30.94%, which fell below the critical threshold of 40%, indicating no significant issue with common method bias.

### Ethics

All research procedures adhered to relevant ethical guidelines and regulations. This study received approval from the medical ethics review board of China Medical University [No.2023–107, Dated 06Mar2023]. Written informed consent was obtained from all participating students, ensuring their voluntary participation and protection of their rights. Informed consent was acquired from all study subjects before their involvement in the research.

## Results

### Participants

A total of 860 questionnaires were distributed using a convenient sampling method, with 852 valid questionnaires successfully recovered, resulting in an effective response rate of 99.07%. The sample consisted of 389 males and 463 females. In terms of academic year, the distribution included 195 students from Grade I, 172 from Grade II, 188 from Grade III, 148 from Grade IV, and 149 from Grade V. Additionally, there were 356 students hailing from urban areas and 496 from rural areas.

### Physical exercise

PARS-3 assessment revealed a mean total score of 28.67 ± 5.54 among medical students. Significant gender differences were observed, with male students demonstrating higher physical activity levels (31.24 ± 7.51) than female students (26.12 ± 4.62), *p* < 0.001, Cohen’s d = 0.87 [95% CI: 0.68–1.06]. This large effect size (d > 0.8) indicates clinically meaningful gender-based activity disparities. Exercise intensity distribution analysis classified participants into three tiers: low-intensity (*n* = 442, 51.9%), moderate-intensity (*n* = 221, 25.9%), and high-intensity (*n* = 189, 22.2%) exercise groups.

### Physical exercise and mental health

Gender-based SCL-90 comparisons showed males scored significantly lower than females in obsessive-compulsive symptoms (1.54 ± 0.42 vs. 1.62 ± 0.37; *p* < 0.001), interpersonal sensitivity (1.49 ± 0.26 vs. 1.58 ± 0.41; *p* < 0.001), depression (1.63 ± 0.22 vs. 1.73 ± 0.34; *p* < 0.001), and anxiety (1.52 ± 0.17 vs. 1.68 ± 0.13; *p* < 0.001). Non-significant differences emerged in somatization, hostility, terror, paranoia, and psychosis (Table 1).

**Table 1:** Psychological symptom scores in SCL-90 (Mean ± SD) by gender.

Factors	Gender		*t*-value	*p*-value
	Male (*n* = 389)	Female (*n* = 463)		
Somatization	1.39 ± 0.18	1.41 ± 0.19	1.498	0.289
Obsession	1.54 ± 0.42	1.62 ± 0.37	4.473	<0.001
Sensitivity	1.49 ± 0.26	1.58 ± 0.41	5.016	<0.001
Depression	1.63 ± 0.22	1.73 ± 0.34	3.532	<0.001
Anxiety	1.52 ± 0.17	1.68 ± 0.13	3.201	<0.001
Hostility	1.56 ± 0.15	1.58 ± 0.16	0.933	0.123
Terror	1.44 ± 0.23	1.49 ± 0.42	1.412	0.063
Paranoia	1.53 ± 0.18	1.60 ± 0.25	1.573	0.098
Psychosis	1.48 ± 0.41	1.52 ± 0.39	1.739	0.106

SCL-90 = Symptom CheckList-90, SD = Standard Deviation. The Student t-test was used for reporting *p*-value and comparing the SCL-90 by gender.

When assessing the impact of physical exercise on mental health, it is observed that medical students engaged in high-and medium-intensity physical exercise had lower scores for depression and psychosis in SCL-90 compared to those engaged in low-intensity exercise (*p* < 0.05). Additionally, students with high-intensity exercise had lower anxiety scores in the SCL-90 than those with medium-intensity exercise (*p* < 0.05). No significant differences were found in the scores for other factors (*p* > 0.05, Table 2). Further analysis revealed a negative correlation between the level of physical activity and the scores for depression (Standard regression coefficient = −0.236, *t* = 3.146, *p* < 0.001) and psychosis in the SCL-90 (Standard regression coefficient = −0.207, *t* = 3.125, *p* < 0.001).

**Table 2:** Comparison of SCL-90-R psychological symptom dimensions across physical activity intensity groups.

Factors	Intensity			*p*-value
	Low (*n* = 442)	Moderate (*n* = 221)	High (*n* = 189)	
Somatization	1.44 ± 0.24	1.42 ± 0.18	1.40 ± 0.16	>0.05
Obsession	1.59 ± 0.52	1.54 ± 0.57	1.52 ± 0.43	>0.05
Sensitivity	1.69 ± 0.34	1.68 ± 0.38	1.64 ± 0.21	>0.05
Depression	1.68 ± 0.22	1.63 ± 0.34	1.53 ± 0.34	0.025*#
Anxiety	1.54 ± 0.19	1.53 ± 0.32	1.44 ± 0.13	0.016*
Hostility	1.62 ± 0.25	1.62 ± 0.09	1.59 ± 0.14	>0.05
Terror	1.55 ± 0.39	1.53 ± 0.37	1.50 ± 0.22	>0.05
Paranoia	1.61 ± 0.36	1.58 ± 0.29	1.57 ± 0.18	>0.05
Psychosis	1.58 ± 0.30	1.52 ± 0.15	1.47 ± 0.27	0.032*#

SCL-90 = Symptom CheckList-90, SD = Standard Deviation. The one-way ANOVO with Scheffe’s post hoc tests was used for reporting p-value and comparing the SCL-90 by different intensity exercise.

^*^Comparison of each factor between low-and high-intensity exercise; #Comparison of each factor between low-and moderate-intensity exercise.

### Physical exercise, emotional regulation, and suicidal ideation

Applying established diagnostic thresholds (NSI ≥ 1.63 and PSI ≤ 3.33) (Lei et al., 2021), 69 students (8.10%) were classified as having active suicidal ideation. No statistically significant differences were observed in suicide ideation scores or total scores concerning gender, grade, or place of origin. After controlling demographic variables such as gender, grade, and birthplace, Table 3 showed a significant positive correlation between physical exercise and emotional regulation (*r* = 0.124, *p* < 0.01), as well as a significant negative correlation between physical exercise and suicidal ideation (*r* = −0.117, *p* < 0.01). Additionally, a significant negative correlation existed between emotional regulation and suicidal ideation (*r* = −0.236, *p* < 0.01). Two-way ANOVA revealed significant main effects of gender on SCL-90 factors and no significant interactions emerged (*p* > 0.05), indicating exercise benefits are gender-invariant (Supplementary Table 1).

**Table 3:** Correlation matrix of physical exercise, emotional regulation, and suicidal ideation.

Pearson correlation coefficients	Physical exercise	Emotional regulation	Suicidal ideation
Physical exercise	1		
Emotional regulation	0.124**	1	
Suicidal ideation	−0.117**	−0.236**	1

** refers to *p*-value <0.01.

### Mediating effect of emotional regulation between physical exercise and suicidal ideation

**Figure 1:**
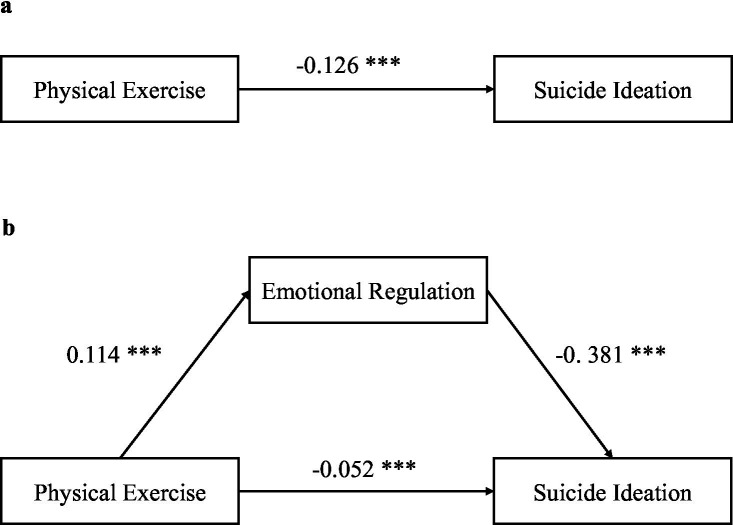
The figure presents two path analysis models examining the relationships between physical exercise, emotional regulation, and suicide ideation. Panel **(a)** shows the direct effect of physical exercise on suicide ideation, with a path coefficient of −0.126* (indicating a significant negative association). Panel **(b)** depicts a mediation model where physical exercise first predicts emotional regulation (path coefficient = 0.114*), which in turn negatively predicts suicide ideation (path coefficient = −0.381*). Additionally, the direct effect of physical exercise on suicide ideation remains significant in the mediation model, with a smaller path coefficient of −0.052*. All reported coefficients are statistically significant at the *p* < 0.001 level (denoted by ***).

Physical exercise had a significant negative predictive effect on suicidal ideation (*β* = −0.126, *p* < 0.001). Emotional regulation also exhibited a positive relationship with physical exercise (*β* = 0.114, *p* < 0.001) (Figure 1a).

In the regression analysis with suicidal ideation as the predictor variable and physical exercise as the outcome variable, physical exercise negatively predicted suicidal ideation (*β* = −0.052, *p* < 0.05). Importantly, emotional regulation was introduced as a mediator, and it significantly negatively predicted suicidal ideation (*β* = −0.381, *p* < 0.001). While the predictive impact of physical exercise on suicidal ideation decreased in the presence of emotional regulation, it remained statistically significant (Figure 1b). This indicates that emotional regulation partially mediated the relationship between physical exercise and suicidal ideation, with the mediating effect accounting for 38.1% of the total effect.

## Discussion

Our study reveals concerningly low physical activity levels among medical students—a global pattern exacerbated by rigorous academic demands that constrain time and energy for exercise (Zhang et al., 2020).

This study demonstrates a significant protective association between physical exercise intensity and reduced psychological distress among Chinese medical students, with emotional regulation mediating this relationship. While contextual factors unique to China’s medical education environment—including the gaokao-driven hyper-competition, extended clinical training periods, and persistent mental health stigma (Li et al., 2021; Liao et al., 2022) —may intensify baseline distress levels, the mechanistic pathways identified possess considerable transferability beyond this specific cohort.

The observed gender disparity in mental health outcomes aligns consistently with global medical trainee studies. Females consistently reported greater psychological vulnerability—a phenomenon documented across U.S. (Dyrbye et al., 2020), German (Walnik et al., 2022), and Saudi medical schools (Abouammoh et al., 2020). Structurally embedded inequities—including clinical training discrimination, unbalanced caregiving duties, and diagnostic biases—drive this gap (Yeung et al., 2023). For Chinese female students specifically, employment pressures and sociocultural expectations compound these issues (Ram et al., 2020), creating a self-perpetuating cycle where reduced exercise participation limits stress coping capacity. Therefore, institutions must prioritize tailored mental health programs offering mentorship, internship access, and flexible exercise scheduling for this demographic.

### Impact of physical exercise on mental health

Exercise intensity demonstrates a dose–response effect on mental health: high/medium-intensity exercisers showed clinically meaningful reductions in depression and psychosis versus low-intensity counterparts, with anxiety relief scaling to intensity levels. These findings suggest that physical exercise plays a beneficial role in promoting the mental health of medical students. Engaging in regular physical activity can enhance self-confidence, improve interpersonal skills, combat loneliness, and bolster students’ perseverance while alleviating academic pressures and preventing negative emotions. Physical exercise serves as a valuable tool for relaxation and mental well-being.

### Mechanisms underlying the relationship between physical exercise and suicidal ideation

Physical exercise reduces suicidal ideation both directly and indirectly: while it alleviates established risk factors like depression and sleep disorders (Lee et al., 2013; Sewall et al., 2020). On a biological level, physical exercise can influence neurotransmitter levels, such as dopamine and serotonin, along with endorphin release, thereby enhancing overall brain function. This, in turn, enables individuals to better regulate negative emotions and foster positive ones, ultimately reducing the risk of suicide (Grasdalsmoen et al., 2020). Additionally, physical exercise’s connection to sleep quality plays a crucial role in its impact on mental health (Vancampfort et al., 2018). Moreover, outdoor exercise within a natural environment, with increased exposure to sunlight, has been associated with improved mental well-being (Corazon et al., 2019).

### Mediating effect of emotional regulation

Our findings confirm that increased physical exercise significantly predicts reduced suicidal ideation among medical students, with intensity-dependent effects demonstrating dose–response characteristics. The establishment of consistent exercise habits emerges as a critical protective factor for mental health, corroborating prior evidence that physical activity reduces suicidality across educational stages (Southerland et al., 2016). Crucially, we observed that: (1) stronger emotional regulation capacity inversely correlates with suicidal ideation, aligning with evidence that maladaptive regulation strategies elevate risk (Law et al., 2015) and (2) emotional regulation partially mediates exercise’s protective effect, explaining its potency as an indirect intervention pathway. This mediation mechanism integrates physiological and psychological benefits into a unified protective framework, substantiating emotion regulation as a primary conduit through which exercise mitigates suicide risk.

### Limitations

Despite our significant findings, this study has limitations. It was conducted at a single medical school in Liaoning Province, potentially limiting the generalizability of our conclusions. While our models controlled for gender, future studies with larger gender-stratified samples could further explore sex-specific patterns in exercise-mediated emotional regulation pathways, particularly given documented disparities in mental health vulnerability and activity patterns. Additionally, due to time and sample size constraints, potential confounding variables, such as peer relationships and the psychological impact of evolving epidemic situations, were not assessed. Future research should expand its scope to encompass a broader range of variables and collect more extensive data to confirm and build upon our results (Jang and Park, 2019).

## Conclusion

This study confirms that insufficient physical activity among Chinese medical students significantly correlates with heightened mental health risks, particularly suicidal ideation. Medical institutions should prioritize structured exercise programs integrated into curricula, with particular emphasis on group-based activities to leverage social support mechanisms. These evidence-based interventions would establish physical activity as a foundational component of mental health infrastructure in high-stress academic environments.

## Data Availability

The original contributions presented in the study are included in the article/Supplementary material, further inquiries can be directed to the corresponding author.
