# Supplementary material for: The impact of physical exercise on mental health and the relationship among physical exercise, emotional regulation and suicidal ideation in Chinese medical students
Source: Front Psychol. 2025 Aug 12;16:1609415. doi: 10.3389/fpsyg.2025.1609415 (PMC12379471; doi:10.3389/fpsyg.2025.1609415)
Supplement: Supplementary file 1 [file Table_1.docx]

Supplementary Table 1. Two-Way ANOVA Results for SCL-90 Psychological Symptom Dimensions by Gender and Exercise Intensity

| **​SCL-90 Factor​** | **​Main Effect: Gender​**  ***F*; *p*; η^2^** | **​Main Effect: Exercise Intensity**  ***F*; *p*; η^2^** | **​Interaction Effect​**  ***F*; *p*; η^2^** | **​Post-Hoc Comparisons​**  **(Tukey HSD)** |
| --- | --- | --- | --- | --- |
| ​**Somatization**​ | *F*=2.24; *p*=.135; η^2^=.003 | *F*=1.87; *p*=.155; η^2^=.004 | *F*=0.92; *p*=.399; η^2^=.002 |  |
| ​**Obsession**​ | *F*=20.01; ***p*<.001**; η^2^=.023 | *F*=8.96; ***p*<.001**; η^2^=.021 | *F*=1.24; *p*=.291; η^2^=.003 |  |
| ​**Interpersonal Sensitivity**​ | *F*=25.16; ***p*<.001**; η^2^=.029 | *F*=11.03; ***p*<.001**; η^2^=.025 | *F*=0.87; *p*=.420; η^2^=.002 |  |
| ​**Depression**​ | *F*=12.48;***p*<.001**; η^2^=.015 | *F*=14.27; ***p*<.001**; η^2^=.032 | *F*=1.05; *p*=.351; η^2^=.002 | **H < L**​ (*p*<.001)  ​**M < L**​ (*p*=.038) |
| ​**Anxiety**​ | *F*=10.25;***p*=.001**; η^2^=.012 | *F*=12.84; ***p*<.001**; η^2^=.029 | *F*=0.96; *p*=.383; η^2^=.002 | ​**H < L**​ (*p*=.002) |
| ​**Hostility**​ | *F*=0.87; *p*=.351; η^2^=.001 | *F*=2.15; *p*=.117; η^2^=.005 | *F*=1.33; *p*=.265; η^2^=.003 |  |
| ​**Phobic Anxiety**​ | *F*=1.99; *p*=.158; η^2^=.002 | *F*=3.01; *p*=.050; η^2^=.007 | *F*=0.75; *p*=.472; η^2^=.002 |  |
| ​**Paranoid Ideation**​ | *F*=2.47; *p*=.116; η^2^=.003 | *F*=4.12; *p*=.017; η^2^=.010 | *F*=1.18; *p*=.309; η^2^=.003 |  |
| ​**Psychoticism**​ | *F*=3.02; *p*=.082; η^2^=.004 | *F*=6.89;***p*=.001**; η^2^=.016 | *F*=0.82; *p*=.442; η^2^=.002 | **H < L**​ (*p*=.007) ​**H < M**​ (*p*=.049) |

​Design: 2 × 3 factorial ANOVA examining effects of ​Gender​ (Male/Female) and ​Exercise Intensity​ (Low/Moderate/High) on SCL-90 symptom dimensions.

​Effect Size: η² = partial eta-squared (small: ≥.01, medium: ≥.06, large: ≥.14 per Cohen, 1988).

​Group Codes: H = High Intensity, M = Moderate Intensity, L = Low Intensity.

​Post-hoc Tests: Tukey HSD comparisons reported only for significant main effects (p < .05).

​Statistical Significance: Bolded p-values indicate p < .05.

​Interaction Interpretation: All interaction effects non-significant (p > .05), indicating ​relationship between exercise intensity and SCL-90 symptom severity did not differ by gender.

​Total Sample: N = 852 medical students.
